# Clinicodemographic aspect of resectable pancreatic cancer and prognostic factors for resectable cancer

**DOI:** 10.1186/1477-7819-10-77

**Published:** 2012-05-04

**Authors:** Kun-Chun Chiang, Chun-Nan Yeh, Shir-Hwa Ueng, Jun-Te Hsu, Ta-Sen Yeh, Yi-Yin Jan, Tsann-Long Hwang, Miin-Fu Chen

**Affiliations:** 1 General Surgery Department, Chang Gung Memorial Hospital, 222, Mai-Chin Road, Keelung 204, Taiwan; 2 Graduate Institute of Clinical Medical Sciences, College of Medicine, Chang Gung University, 259 Wen-Hwa 1st Road, Kwei-Shan, Tao-Yuan 333, Taiwan; 3 General Surgery Department, Chang Gung Memorial Hospital, Fu-Hsing Street, Kwei-Shan, Taoyuan 333, Taiwan; 4 Department of Pathology, Chang Gung Memorial Hospital, Fu-Hsing Street, Kwei-Shan, Taoyuan 333, Taiwan; 5 Department of Surgery, Fu-Hsing Street, Kwei-Shan, Taoyuan, Taiwan

## Abstract

**Background:**

Pancreatic adenocarcinoma (PCA) is one of the most lethal human malignancies, and radical surgery remains the cornerstone of treatment. After resection, the overall 5-year survival rate is only 10% to 29%. At the time of presentation, however, about 40% of patients generally have distant metastases and another 40% are usually diagnosed with locally advanced cancers. The remaining 20% of patients are indicated for surgery on the basis of the results of preoperative imaging studies; however, about half of these patients are found to be unsuitable for resection during surgical exploration. In the current study, we aimed to determine the clinicopathological characteristics that predict the resectability of PCA and to conduct a prognostic analysis of PCA after resection to identify favorable survival factors.

**Methods:**

We retrospectively reviewed the medical files of 688 patients (422 men and 266 women) who had undergone surgery for histopathologically proven PCA in the Department of Surgery at Chang Gung Memorial Hospital in Taiwan from 1981 to 2006. We compared the clinical characteristics of patients who underwent resection and patients who did not undergo resection in order to identify the predictive factors for successful resectability of PCA, and we conducted prognostic analysis for PCA after resection.

**Results:**

A carbohydrate antigen 19–9 (CA 19–9) level of 37 U/ml or greater and a tumor size of 3 cm or more independently predicted resectability of PCA. In terms of survival after resection, PCA patients with better nutritional status (measured as having an albumin level greater than 3.5 g/dl), radical resection, early tumor stage and better-differentiated tumors were associated with favorable survival.

**Conclusions:**

Besides traditional imaging studies, preoperative CA 19–9 levels and tumor size can also be used to determine the resectability of PCA. Better nutritional status, curative resection, early tumor stage and well-differentiated tumors predict the favorable prognosis of PCA patients after resection.

## Background

Pancreatic adenocarcinoma (PCA) is one of the most lethal human malignancies and ranks as the eighth and ninth most common causes of cancer-related mortality worldwide for men and women, respectively [1]. In the United States in 2008, 37,680 new cases of PCA were diagnosed, and 34,290 PCA-related deaths occurred [2]. The incidence-to-mortality ratio was nearly 1:1, illustrating the lethality of PCA. The overall 5-year survival rate of patients with PCA is estimated to be approximately 1% to 4%, a percentage range that is likely related to the aggressive characteristics of PCA, such as early local spread and metastasis and resistance to radiotherapy and most systemic chemotherapies [3]. Currently, radical surgical resection is the cornerstone of treatment. After resection, the overall 5-year survival rate is only 10% to 29% [4-6]. However, previous studies have shown that, at the time of presentation, about 40% of patients have distant metastases and another 40% are diagnosed with locally advanced cancers [2,3,7]. The remaining 20% of patients are indicated for surgery on the basis of the results of preoperative imaging studies; however, about half of these patients are found to be unsuitable for resection during surgical exploration [8-12]. Unnecessary surgical exploration may, in turn, lead to increased surgical risk and healthcare costs and may delay systemic treatment [13].

Therefore, accurate preoperative prediction of PCA resectability is crucial to facilitating appropriate management of PCA patients. In the past few years, researchers have attempted to address this issue and have found that preoperative measurement of carbohydrate antigen 19–9 (CA 19–9) level, endoscopic ultrasonography (EUS), computed tomography (CT) and staging laparoscopy may enhance the accuracy of prediction of resectability before surgery [14-18].

Herein we retrospectively review the medical files of 688 PCA patients who underwent surgery at our hospital from 1981 to 2006 and identified the predictive factors for resectability of PCA by comparing the clinical characteristics of patients who underwent resection with those of patients who did not undergo resection. Furthermore, we also investigated the prognostic factors for favorable PCA outcomes following resection.

## Methods

We retrospectively reviewed the medical files of 688 patients who had undergone surgery for histopathologically proven PCA in the Department of Surgery at Chang Gung Memorial Hospital in Taipei, Taiwan, from 1981 to 2006. This retrospective study was approved by the local institutional review board of Chang Gung Memorial Hospital (clinical study no. 94-955B).

Resection was defined as pancreatectomy (Whipple operation or distal pancreatectomy), regardless of the status of pancreatic resection margin. The patients comprised 422 men and 266 women with a median age of 64 years (range, 14 to 93 years). Of these patients, 230 underwent resection (the resection group), and the remaining 458 patients were unable to undergo resection because of portal vein invasion or carcinomatosis (the no-resection group). Surgical mortality was defined as death within 1 month of surgery. Laboratory tests were conducted 1 day before surgery. Serum CA 19–9 and carcinoembryonic antigen (CEA) levels were measured by radioimmunoassay. Tumors were preoperatively evaluated by abdominal ultrasonography, endoscopic retrograde cholangiopancreatography (ERCP), percutaneous transhepatic cholangiography (PTC), CT, magnetic resonance imaging with cholangiopancreatography (MRCP) and angiography, as appropriate. Tumor stage was defined according to the pathological tumor node metastasis (pTNM) classification proposed by the Union for International Cancer Control (UICC). Stages I and II represent early-stage PCA, and stages III and IV represent advanced-stage PCA. Patients with a tumoral resection margin or lymph node metastasis were given adjuvant chemotherapy consisting of a systemic 5-fluorouracil- or gemcitabine-based regimen. Adjuvant radiotherapy was conducted by intraoperative radiotherapy, external beam radiotherapy and/or brachytherapy in patients with either a positive section margin or local recurrence. The follow-up period ranged from 1 to 169.4 months, and, during follow-up, abdominal CT, chest X-ray, MRCP and tumor marker measurement were performed as appropriate. In terms of tumor size, we chose 3 cm as the cutoff value to investigate its impact on the resectablity of pancreatic cancer based on our own experience.

### Statistical analysis

All data are presented as the proportion (%) of patients or as means with standard deviations. Numerical data were compared using independent Student’s *t*-tests. Nominal data were compared using Pearson’s χ^2^ test or forward stepwise multiple logistic regression, as appropriate. Survival rates were calculated and plotted using the Kaplan-Meier method. Clinicopathological variables, including demographic data, laboratory data, clinical features, pathological features and operative findings, were selected for survival analysis. We performed survival analysis using the logrank test and multivariate analysis using the Cox proportional hazards model. All statistical analyses were performed using the SPSS computer software package (version 10.0; Chicago, IL, USA). A *P*-value less than 0.05 was considered statistically significant.

## Results

### Survival of PCA patients

**Figure 1:**
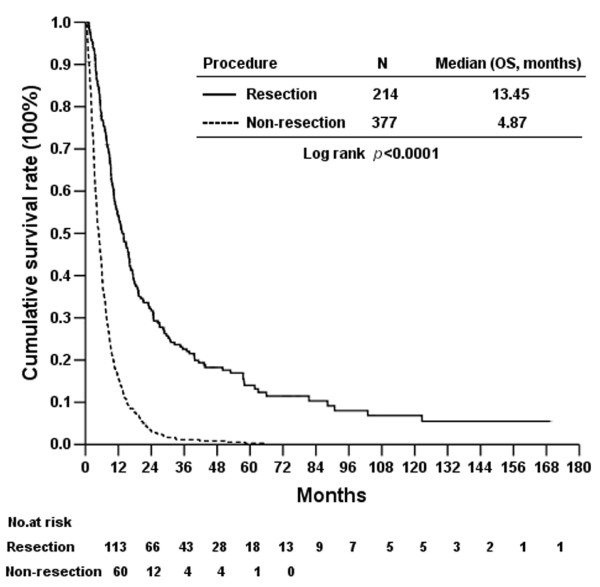
The difference of overall survival rates between 214 pancreatic cancer patients who underwent pancreatic resection and 377 pancreatic cancer patients who did not undergo pancreatic resection.

The median follow-up time for all patients in this study was 6.5 months (range, 1 to 169.4 months). For the resection group, the median follow-up time was 13 months (range, 1 to 169.4 months) and, for the no-resection group, the median follow-up time was 4.9 months (range, 1 to 65.7 months). Ninety-seven patients were excluded from the survival analysis because of lack of follow-up data or death within 1 month of surgery. The overall 1-, 3- and 5-year survival rates for the remaining 591 PCA patients were 29.7%, 8.8% and 5.1%, respectively. Patients in the resection group showed significantly better survival than patients in the no-resection group (Figure 1). The overall 3-year survival rate was significantly higher in the resection group (22.6%) than in the no-resection group (1.1%) (*P* < 0.0001). In addition, the PCA resection rate has increased significantly in our hospital over the past 3 decades, from, respectively, 16% to 27.7% to 48.8% for 1981 to 1999, 1991 to 2000 and 2001 to 2006 (*P* < 0.0001).

### Predictive factors for resectability of PCA

Table 1 summarizes the clinicopathological features of the 688 PCA patients. There were 230 patients in the resection group and 458 patients in the no-resection group. The mean age in the resection group was significantly lower than that in the no-resection group (*P* = 0.047), and the resection group had a greater proportion of patients ages 65 years or younger (*P* = 0.015). The gender ratio in the two groups was similar. No differences were observed between the two groups with regard to preoperative physical examination findings, but patients in the no-resection group tended to be asymptomatic before the operation (*P* = 0.018). Tumor distribution (head or of the pancreas or not) was also similar in the two groups. Patients in the resection group showed higher albumin levels than those in the no-resection group (3.77 ± 0.65 g/dl versus 3.63 ± 0.17 g/dl; *P* = 0.025). Furthermore, the proportion of patients with albumin levels greater than 3.5 g/dl in the resection group was higher than that in the no-resection group (*P* = 0.02). The average of preoperative aspartate aminotransferase (AST) level was higher in the resection group than in the no-resection group. The bilirubin levels were similar in both groups. With regard to the serum levels of the tumor marker CA 19–9, we found lower mean CA 19–9 levels in the resection group than in the no-resection group, but the difference was not statistically significant. In addition, the proportion of patients with CA 19–9 levels above 37.0 U/ml in the no-resection group was higher than that in the resection group (*P* = 0.007). Similarly, CEA levels were higher in the no-resection group, and a higher proportion of patients in the no-resection group had CEA levels greater than 5 ng/ml. The mean tumor size in the no-resection group was 6.2 ± 3.5 cm, and that in the resection group was 4.61 ± 3.6 cm. Because we had set 3 cm as the cutoff value for tumor size, the no-resection group obviously showed a higher rate of tumors over 3 cm than the resection group. Moreover, the no-resection group had shorter operation times, fewer postoperative complications and lower mortality rates than the resection group did.

**Table 1:** Characteristics of pancreatic ductal adenocarcinoma in patients with either resectable or nonresectable tumors

**Characteristics**	**Resectable tumor (*n*= 230)**	**Non-resectable tumor (*n*= 458)**	***P*value**
Age (years)	62.1 ± 11.1	64.0 ± 12.0	0.047
<65	136 (59.1%)	226 (49.3%)	0.015
≥65	94 (40.9%)	232 (50.7%)	
Sex			0.749
Male	143(62.2%)	279 (60.9%)	
Women	87 (37.8%)	179 (37.1%)	
Symptoms			0.018
Yes	225 (97.8%)	457 (99.8%)	
No	5 (2.2%)	1 (0.2%)	
Physical findings			0.979
Yes	166 (72.2%)	331 (72.3%)	
No	64 (27.8%)	127 (27.7%)	
Bilirubin (mg/dl)	7.5 ± 9.1	7.2 ± 8.5	0.574
≤1.3	80 (36.5)	177 (42.3)	0.155
>1.3	139 (63.5)	241 (57.7)	
Albumin (g/dl)	3.77 ± 0.65	3.63 ± 0.71	0.025
≤3.5	61 (33.0%)	149 (43.3%)	0.020
>3.5	124 (67.0%)	195 (56.7%)	
AST (U/L)	112.9 ± 146.8	84.8 ± 99.2	0.011
≤68	110 (49.1%)	250 (60.1%)	0.008
>68	114 (50.9%)	166 (39.9%)	
CEA (ng/ml)	6.1 ± 8.1	13.5 ± 48.0	0.008
≤5	115 (67.6%)	180 (58.4%)	0.047
>5	55 (32.4%)	128 (41.6%)	
CA 19–9 (U/ml)	1,229.6 ± 4,680.4	1,616.4 ± 5,253.1	0.425
≤37	48 (27.1%)	46 (16.5%)	0.007
>37	129 (72.9%)	232 (83.5%)	
Operation time (minutes)	426.0 ± 155.3	219.2 ± 172.4	<0.0001
≤240	31 (13.9%)	285 (68.3%)	<0.0001
>240	192 (86.1%)	132 (31.7%)	
Complication			0.003
Yes	55 (23.9%)	67 (14.6%)	
No	175 (76.1%)	391 (85.4%)	
Death			0.017
Yes	7 (3.0%)	36 (7.6%)	
No	223 (97.0%)	423 (92.4%)	
Tumor size (cm)	4.6 ± 3.6	6.2 ± 3.5	<0.0001
≤3	94 (42.7%)	24 (15.8%)	<0.0001
>3	126 (57.3%)	128 (84.2%)	
Location			0.111
Head of pancreas	165 (71.7%)	301 (65.7%)	
Not at head of pancreas	65 (28.3%)	157 (34.3%)	
Differentiation			0.0002
Well	83 (36.1%)	134 (29.3%)	
Moderate	105 (45.7%)	173 (37.8%)	
Poor	35 (15.2%)	103 (22.5%)	
Others	7 (3.0%)	48 (10.5%)	
Post-op C/T			<0.0001
Yes	121 (52.6%)	163 (35.6%)	
No	109 (47.7%)	295 (64.4%)	
Post-op R/T			0.255
Yes	17 (7.4%)	46 (10.0%)	
No	213 (92.6%)	412 (90.0%)	

AST: aspartate aminotransferase; CEA: carcinoembryonic antigen; CA 19–9: carbohydrate antigen 19–9; Post-op C/T: postoperative chemotherapy; Post-op R/T: postoperative radiotherapy; Others: mixed tumor differentiation.

All significant predictive factors obtained in the univariate analysis (Table 1) were included in our multivariate analysis, which was performed using a logistic regression method. Only CA 19–9 level <37 and tumor size <3 cm were independent predictive factors for resectability of PCA.

#### Factors influencing the survival of PCA patients after resection

Univariate analysis (Table 2) indicated that women had a better prognosis than men (*P* = 0.02), with overall 3- and 5-year survival rates of 29.9% and 23.1%, respectively, for women and 3- and 5-year overall survival rates of 18.3% and 8.6%, respectively, for men. An albumin level greater than 3.5 g/dl was a favorable prognostic factor for survival after PCA resection (*P* = 0.024). Other factors, such as nontumoral resection margin, bilirubin levels of 1.3 mg/dl or less, CA 19–9 levels of 37 U/ml or less, no lymph node metastasis, early pTNM stage and well-differentiated tumors, were also associated with a better prognosis. On the other hand, age, preoperative physical examination findings and preoperative biochemical data (including levels of amylase, lipase and the tumor marker CEA) were not associated with patient survival after resection of PCA. Interestingly, we found that tumor location, operative procedure and portal vein resection were not related to patient survival after PCA resection.

**Table 2:** Univariate analysis of factors influencing the overall survival of the 214 PAC patients

**Factors**	**Data (*n*)**	**Median survival (months)**	**95% CI of median**	**1 year**	**3 years**	***P*-value**
Age (years)						0.068
	≤65 (130)	16.08	13.23 to 18.93	26.6	15.3	
	>65 (26)	10.28	9.05 to 11.53	16.8	12.0	
Sex						0.020
	Male (125)	11.18	8.51 to 13.85	18.3	8.6	
	Female (89)	16.57	14.05 to 19.09	29.9	23.1	
Symptoms						0.007
	Positive (209)	13.05	10.30 to 15.50	21.2	12.4	
	Negative (5)	NA		80.0	80.0	
Bilirubin (mg/dl)						0.009
	≤1.3 (73)	17.39	12.19 to 22.59	26.7	26.9	
	>1.3 (130)	12.69	10.14 to 15.24	20.9	8.2	
Albumin (g/dl )						0.024
	≤3.5 (54)	9.04	5.50 to 12.58	15.2	10.2	
	>3.5 (117)	15.75	12.45 to 19.05	25.4	16.7	
Amylase (U/L)						0.216
	≤300 (82)	13.05	8.77 to 17.33	19.3	13.4	
	>300 (12)	19.27	0.00 to 63.26	50.0	10.0	
Lipase (U/L)						0.255
	≤300 (47)	12.26	8.48 to 16.05	13.4	8.9	
	>300 (30)	16.04	6.03 to 26.05	26.7	16.0	
CEA (ng/ml)						0.159
	≤5 (109)	15.85	12.19 to 19.52	23.8	17.5	
	>5 (52)	10.42	7.04 to 13.80	24.7	6.1	
CA 19–9 (U/ml)						0.044
	≤37 (44)	19.43	8.50 to 30.37	32.6	22.3	
	>37 (125)	13.05	10.08 to 16.02	19.7	9.6	
Surgical procedure						0.115
	Whipple (119)	12.79	9.90 to 15.69	21.2	10.8	
	PPPD (39)	13.05	7.25 to 18.85	23.7	13.5	
	Others (56)	17.19	11.81 to 22.57	25.1	25.1	
Resection margin (cm)						0.001
	Nontumoral	15.42	13.12 to 17.72	58.8	27.3	
	Tumoral	9.67	8.15 to 11.19	42.1	10.5	
Tumor location						0.096
	Head (152)	12.79	10.34 to 15.24	21.3	11.12	
	Uncinate process (20)	19.13	3.79 to 34.47	32.8	32.8	
	Body (32)	19.27	9.75 to 28.80	22.7	22.7	
	Tail (10)	8.19	2.18 to 14.20	20.0	NA	
Portal vein resection						0.249
	Performed (12)	9.27	0.00 to 19.54	16.7	0.0	
	Not performed (202)	13.45	10.62 to 16.29	23.0	15.1	
Tumor size (cm)	<3 (90)	11.89	11.89 to 17.83	24.6	12.7	0.874
	>3 (116)	8.02	8.02 to 16.30	21.9	14.5	
Nodal status	Negative (108)	18.87	14.04 to 23.70	34.0	23.1	<0.0001
	Positive (105)	9.4	7.98 to 10.82	11.1	4.7	
TNM staging	I (32)	62.99	18.01 to 107.97	56.7	51.0	<0.0001
	II (76)	15.78	12.77 to 18.79	24.9	12.3	
	III (106)	9.4	7.98 to 10.82	11.1	4.7	
Tumor differentiation	Well (76)	26.4	21.09 to 31.77	39.5	28.3	<0.0001
	Moderate (98)	10.32	8.39 to 12.25	14.0	7.9	
	Poor (35)	9.47	5.67 to 13.27	12.1	3.0	
	Undifferentiated (5)	5.46	4.60 to 6.32	40.0	0.0	
Post-op radiotherapy						0.849
	Performed (17)	17.56	10.45 to 24.67	17.6	11.8	
	Not Performed (197)	12.79	9.79 to 15.80	23.1	14.3	
Post-op chemotherapy						
	Performed (119)	14.93	11.70 to 18.16	22.7	10.4	
	Not Performed (95)	10.32	7.44 to 13.20	22.5	19.5	

CEA: carcinoembryonic antigen; CA 19–9: carbohydrate antigen 19–9; NA: not applicable; TNM: tumor, node, metastasis; PPPD: pylorus preserving pancreaticoduodenectomy.

**Figure 2:**
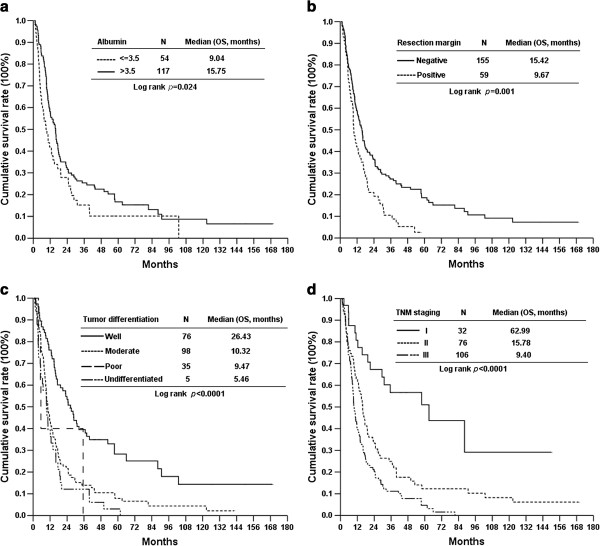
The difference of overall survival rates between 214 pancreatic cancer patients undergoing pancreatic resection in terms of (a) albumin, (b) resection margin status, (c) tumor differentiation and (d) tumor staging.

In the analysis of the significant factors using the multivariate Cox proportional hazards method, we identified albumin levels greater than 3.5 g/dl (Figure 2a), nontumoral resection margin (Figure 2b), well-differentiated tumors (Figure 2c) and early pTNM stage (Figure 2d) as favorable prognostic factors after resection of PCA.

## Discussion

Pancreatic cancer ranks as the fifth most common cause of cancer-related mortality worldwide, and patients with PCA generally have a very poor prognosis [3]. Radical surgical resection is the most effective treatment for PCA, because pancreatic tumors tend to respond poorly to both radiotherapy and chemotherapy. According to the results of previous studies, however, only 20% of PCA patients are considered suitable for resection prior to surgical exploration, and during surgical exploration, half of these patients are found to be unsuitable for resection because of the advanced stage of the tumor [7].

Recent improvements in radiological imaging techniques have decreased the rate of unnecessary surgical exploration; however, the current state of PCA resectability prediction remains unsatisfactory [19-21]. Thus far the method of choice for diagnosing and staging PCA is thin-section, contrast-enhanced, triple-phase CT [22,23]. This method can result in a 90% to 100% predictive value for the unresectability of PCA [14,24-26], but only a 52% to 96% predictive value for resectability has been reported [8,11,24,27,28]. Other methods, such as EUS or 2-^18^F]-fluoro-2-deoxy-d-glucose positron emission tomography (FDG-PET) is also unable to provide satisfactory predictive value regarding resectablity before surgery [16,29-32]. Taken together, these studies demonstrate that no imaging studies can yet provide a complete picture of the preoperative status of PCA to clinical physicians, and thus further investigation of potential ancillary predictive factors is warranted.

In this study, we identified the clinicopathological features of PCA patients that may be useful for predicting the resectability of PCA. Physicians can use these ancillary predictive factors, in combination with traditional imaging studies, to determine the resectability of PCA with increased accuracy.

In the current study, patients in the resection group were, on average, 2 years younger than those in the no-resection group (62 versus 64 years old; *P* = 0.047), and the resection group comprised a greater proportion of patients ages 65 years or younger (*P* = 0.015). This observation suggests that there may be a 2-year period during which PCA progresses from being resectable to becoming unresectable. We also found that CA 19–9 level 37 U/ml or less and tumor size 3 cm or less were independent factors predicting the resectability of PCA. Furthermore, patients in the resection group had longer operation times. The results of our study suggest that both CA 19–9 (≤37 U/ml) and tumor size (≤3 cm) could be used as ancillary parameters to determine the resectability of PCA (odds ratios, 2.458 and 3.155, respectively).

Serological markers of malignancy are widely used as adjuncts to the results of imaging studies for diagnosing malignancy and predicting prognosis. CA 19–9, initially detected by Koprowaki *et al*. [33], is the most well-established tumor marker for PCA, with higher sensitivity and specificity than CEA, CA 50, and CA 242 [34-36]. Some recent studies have emphasized the importance of preoperative CA 19–9 levels in determining the resectability of PCA [15,37]. Maithel *et al*. recommended staging laparoscopy for patients whose CA 19–9 levels exceeded 130.0 U/ml and who were diagnosed with resectable PCA as indicated by radiography [17]. In our study, a lower percentage of patients in the resection group had preoperative CA 19–9 levels greater than 37 U/ml, while CEA levels failed to independently predict the resectability of PCA, an observation that is in line with previous reports [34-36]. Although many studies have investigated the relationship between tumor size and prognosis in PCA [38-40], few studies have associated tumor size with resectability. In the current study, we found that tumor size (cutoff value, 3 cm) could be used to predict the resectability of PCA independently.

For decades, researchers have attempted to determine the factors predictive of favorable outcomes after resection of PCA. In contrast to the study conducted by Schmidt *et al*., our present study did not show that CEA level could be used as a prognostic factor for PCA following tumor resection [41]; however, researchers in other studies have reported findings similar to ours [38,42]. CA 19–9 level has been widely reported to be a prognostic factor for PCA after tumor resection [40,43,44]. Schmidt *et al*. [41] also demonstrated that increased bilirubin predicted unfavorable survival outcomes after PCA resection. In our study CEA, however, neither CA 19–9 nor bilirubin independently indicated a worse outcome after PCA resection. Interestingly, in addition to well-established indicators of survival, such as resection margin status, tumor stage and histological differentiation, we identified serum albumin as an independent prognostic factor after resection of PCA in our study. Albumin has previously been shown to be related to the prognosis of cancer patients [45,46]. In pancreatic cancer, Ruiz-Tovar *et al*. reported that the preoperative serum albumin level (with the cutoff value set at 2.8 g/dl) could be used as a prognostic factor for PCDA [47]. Malnutrition has long been deemed a severe problem involving complex mechanisms in cancer patients [48]. The lower serum albumin concentration in advanced cancer patients may be due to the release of some cytokines such as interleukin 6 or TNF, to suppression of hepatocyte production of albumin, or to increased capillary permeability to albumin by the tumor or its surrounding tissues [46,49]. For example, in conditions of liver metastasis existing, Kupffer cells in the liver would be stimulated to produce interleukin 6 and TNF. Taken together, serum albumin could be deemed a good indicator of cancer survival. In this study, albumin levels greater than 3.5 g/dl were found to be associated with a better prognosis for PCA survival after resection.

## Conclusion

On the basis of our study results, we propose that preoperative CA 19–9 levels and tumor size less than 3 cm can be used as auxiliary parameters, in combination with traditional imaging studies, to determine the resectability of PCA. Furthermore, albumin levels, resection margin status, tumor stage and histological differentiation can be used as prognostic factors for survival after resection of PCA.

### Consent statement

This study has been approved by Chang Gung memorial hospital IRB board. The approved IRB number is 94-955B. A copy of the approval of IRB is available for review by the Editor-in-Chief of this journal.

## Abbreviation

TNF: Tumor necrosis factor.

## Competing interests

The authors declare that they have no competing interests.

## Authors’ contributions

K-CC wrote the manuscript, S-HU carried out the pathological examination, J-TH helped write the manuscript, T-SY helped write the manuscript,Y-YJ participated in data collection, T-LH helped write the manuscript, M-FC helped review this manuscript. C-NY finalized the manuscript. All authors read and approved the final manuscript.

## Foundation

This work was supported by Chang Gung Medical Research Program (CMRP) grant 280271G and 280272G to Dr. Kun-Chun Chiang.
